# Pasuchaca (*Geranium dielsiaum* Knuth): A New Source of Astilbin with Antiglycation Activity

**DOI:** 10.3390/foods14234167

**Published:** 2025-12-04

**Authors:** Guanglei Zuo, Zhaoyang Wu, Hyun-Yong Kim, Jinghui Feng, Soo Kyeong Lee, Yanymee Nimesia Guillen Quispe, Soon Sung Lim

**Affiliations:** 1 Pharmaceutical Informatics Institute, College of Pharmaceutical Sciences, Zhejiang University, Hangzhou 310058, China; 2 Jinhua Institute of Zhejiang University, Zhejiang University, Jinhua 321002, China; 3 College of Medicine, Jinhua University of Vocational Technology, Jinhua 321007, China; 4 Department of Food Science and Nutrition, Hallym University, Chuncheon 24252, Republic of Korea; wzy19970202@163.com (Z.W.); khy9514@kiom.re.kr (H.-Y.K.); ilove0977@nate.com (S.K.L.); 5 College of Pharmaceutical Engineering, Jinhua University of Vocational Technology, Jinhua 321016, China; 20221099@jhc.edu.cn; 6 Institute of Korean Nutrition, Hallym University, Chuncheon 24252, Republic of Korea; 7 Department of Molecular Medicine and Biopharmaceutical Sciences, Graduate School of Convergence Science and Technology, Seoul National University, Seoul 08826, Republic of Korea; estreyany@gmail.com

**Keywords:** Pasuchaca, *Geranium dielsiaum* Knuth, antiglycative activity, MGO-HPLC, HSCCC, astilbin

## Abstract

Pasuchaca (*Geranium dielsianum* Knuth), a traditional Peruvian medicinal plant from the Geraniaceae family used for diabetes management, was investigated for its antiglycative properties. This study aimed to screen, isolate, and identify the active antiglycative compounds from its aerial parts. By coupling a methylglyoxal (MGO)-HPLC screening assay with high-speed counter-current chromatography (HSCCC), seven dihydroflavonol derivatives were separated and identified from the 80% methanol extract. The compounds were identified as 2,3-dihydromyricetin 3-*O*-*α*-rhamnopyranoside (**1**), (+)-taxifolin 3-*O*-*β*-D-xylopyranoside (**2**), astilbin (**6**), isoastilbin (**8**), 3″-acetyl astilbin (**9**), and 2″-acetyl astilbin (**11**). Astilbin was identified as the major constituent, with remarkably high contents of 252.41 mg/g in the 80% methanol extract and 541.04 mg/g in the partitioned upper layer fraction. Astilbin demonstrated potent antiglycation activity across all stages of protein glycation (early, middle, late, and whole stages), significantly surpassing the positive control aminoguanidine. Furthermore, the formation of MGO-astilbin adducts was confirmed by LC-ESI-MS, validating its role as an effective MGO scavenger. This report is the first to isolate these phytochemicals from Pasuchaca. The findings establish astilbin as the key antiglycative component of Pasuchaca, substantiating its traditional use and highlighting its potential as a source of functional food ingredients or natural therapeutics for mitigating glycative stress.

## 1. Introduction

Advanced glycation end products (AGEs) are a diverse group of compounds formed via non-enzymatic reactions between reducing sugars and proteins, nucleic acids, or lipids, thereby altering their structural and functional integrity [1]. These modifications underpin the pathogenesis of various diseases, including diabetes complications, inflammatory arthritis, and cardiovascular and neurodegenerative disorders [1,2,3]. Under hyperglycemic conditions, AGE formation is markedly accelerated, with methylglyoxal (MGO) being the most critical precursor [2]. MGO itself is implicated in type 2 diabetes, cardiovascular diseases, neurodegenerative diseases, aging, and other chronic inflammatory diseases [4,5,6,7,8]. Given the key role of MGO in AGE generation, its scavenging presents an effective strategy to inhibit AGE formation [9,10]. Consequently, targeting the AGEs and MGO has emerged as a promising therapeutic approach for preventing and treating a range of related diseases, including diabetes [5,11,12,13].

The MGO–HPLC assay serves as an effective method for rapidly screening MGO scavengers, such as polyphenols, from natural products, as these compounds can form adducts with MGO, altering their HPLC retention behavior [14,15]. Meanwhile, High-Speed Counter-Current Chromatography (HSCCC) is a unique separation technique based on continuous liquid–liquid partitioning, which efficiently isolates target compounds when a suitable solvent system is employed [16]. Therefore, coupling the MGO-HPLC assay with HSCCC is expected to significantly enhance the efficiency of screening and isolating antiglycative compounds from natural sources.

Pasuchaca (*Geranium dielsianum* Knuth, synonym *Geranium ruizii* Hieron.), a perennial plant native to Peru, is traditionally used in South America to treat diabetes, inflammation, and other ailments [17,18,19]. Local communities commonly consume its decoction as a tea for diabetes prevention [18]. Scientific investigations have corroborated its potential, revealing strong antiglycative activity in vitro [17], alongside other biological effects such as *α*-glucosidase inhibition, antioxidant and hypoglycemic properties, and modulation of the gut environment [18,19,20,21,22,23]. Industrial processing studies have further demonstrated its potential for producing nutraceutical products, such as wholemeal flour, filter infusion, and a functional additive, with the stem exhibiting particularly high levels of total phenolic content (267.66 mg Gallic Acid Equivalent/g dry mass) and antioxidant activity (12.7 mM Trolox Equivalent/g dry mass) [24]. Despite this evidence, until recently, the specific phytochemicals of Pasuchaca remain unidentified, which significantly limits its further development and quality control. Therefore, this study aimed to efficiently screen, isolate, and identify the antiglycative compounds in Pasuchaca by integrating the MGO-HPLC assay for rapid screening with HSCCC for efficient isolation.

## 2. Materials and Methods

### 2.1. Materials

All chemicals and solvents were acquired from commercial sources. Analytical grade solvents for extraction and HSCCC were provided by Samchun Pure Chemical (Pyeongtaek-si, Republic of Korea), while HPLC-grade methanol was from J.T. Baker (Phillipsburg, NJ, USA). The MGO solution (40% in H_2_O), aminoguanidine hydrochloride (AG), *o*-phenylenediamine (1,2-diaminobenzene), fructose, D-ribose, trifluoroacetic acid (99%, TFA), sodium azide, 1,2-diaminobenzene, glacial acetic acid, and various inorganic salts were purchased from Sigma-Aldrich (Saint Louis, MO, USA). Additionally, we sourced 4-Nitro blue tetrazolium chloride from Roche Diagnostics (Mannheim, Germany), the G.K. peptide from BACHEM (Bubendorf, Switzerland), Bovine serum albumin (BSA) from Bovogen Biologicals (Keilor East, Australia), and Sephadex™ LH-20 from GE Healthcare (Chicago, IL, USA). All aqueous solutions were prepared using ultra-pure water from a Milli-Q system (Molsheim, France).

The research material, dried aerial parts of Pasuchaca, was obtained from Lima, Peru. The identity of the plant was confirmed by Prof. Paul H. Gonzales Arce at the Museo de Historia Natural, Peru, and a representative specimen has been archived at Hallym University, Korea.

### 2.2. Extraction

For the comparative extraction, dried aerial parts of Pasuchaca (1 g each) were extracted three times with 10 mL of methanol-water solutions at varying concentrations (0%, 20%, 40%, 60%, 80%, and 100% MeOH). The extractions were performed at 37 °C for 12 h in a shaking incubator (120 rpm; Sejong Scientific Co., Gyeonggi-do, Republic of Korea). The respective extracts were then combined, filtered, analyzed by HPLC (injection volume: 10 μL), and concentrated to dryness under reduced pressure.

For large-scale preparation, 150 g of the dried aerial parts was extracted three times with 1.5 L of 80% aqueous methanol under the same conditions (37 °C, 120 rpm, 12 h). The combined extracts were filtered through a 5 μm membrane (Advantec #2) and evaporated to dryness using a rotary evaporator at 37 °C, yielding 51.51 g of the 80% MeOH extract, which corresponds to an extraction yield of 34.34% (*w*/*w*).

### 2.3. HPLC Analysis

HPLC analyses were conducted on Thermo LCQ Advantage or Dionex systems equipped with an Agilent ZORBAX 300SB-C18 column (3.0 × 150 mm, 3.5 μm; 30 °C). Samples (5–15 μL) were eluted at 0.7 mL/min with a gradient of MeOH (B) in 0.1% formic acid (A): 5–32% B (0–8 min), 32–35% B (8–13 min), 35–50% B (13–16 min), 50–100% B (16–20 min), held at 100% B (20–23 min), returned to 5% B (23–25 min), and re-equilibrated (25–30 min). Detection was at 280 nm. The Dionex system was used for analyzing Sephadex/HSCCC fractions and astilbin quantification; all other analyses used the Thermo system.

### 2.4. Antiglycation Assay

All antiglycation assays were performed in triplicate, and the results are presented as mean ± standard deviation (SD). The detailed procedures for each assay are described below.

#### 2.4.1. BSA-Fructose Assay

The antiglycative activity during the entire glycation process was assessed by a BSA-fructose assay [9]. In brief, 400 μL of BSA solution (50 mg/mL in 0.1 M PBS at pH 7.4, with 0.02% sodium azide) was mixed with 200 μL of sample (extracts 125–500 μg/mL, pure compounds 312.5–1250 μM; prepared in water) and pre-incubated at 50 °C for 10 min. After adding 400 μL of fructose solution (1.25 M in 0.1 M PBS at pH 7.4), the mixture was incubated at 50 °C for 24 h. Then, 200 μL of the reaction mixture was analyzed for AGE-associated fluorescence (*λ*_ex_ = 360 ± 20 nm, *λ*_em_ = 460 ± 20 nm) using a BioTek FL × 800 microplate reader (BioTek, Winooski, VT, USA). AG (50–200 μg/mL or 500–2000 μM) served as the positive control. The percentage inhibition of protein glycation was calculated using Equation (1),

(1) $$\%\mathrm{inhibition}=\left(1-\frac{f_{sample}-f_{blank1}}{f_{control}-f_{blank2}}\right)\times 100\%,$$

where *f_control_* is the fluorescence intensity of fructose-mediated AGEs without sample; *f_sample_* is the fluorescence intensity of fructose-mediated AGEs treated with sample; *f_blank_*_1_ is the fluorescence intensity of the mixture of BSA and sample, but without fructose; *f_blank_*_2_ is the fluorescence intensity of BSA alone (without fructose or sample).

#### 2.4.2. Determination of Fructosamine

The formation of fructosamine, an early-stage glycation product, was quantified using the nitroblue tetrazolium (NBT) assay [25]. The glycation solutions were prepared as described in Section 2.4.1. For the NBT assay, a mixture of 35 μL of water, 160 μL of 0.3 mM NBT solution (in 0.1 M sodium carbonate buffer, pH 10.35), and 5 μL of either water (blank group), sample-absent glycation solution (control group), or sample-containing glycation solution (sample group) was combined in a 96-well plate. After the 25 °C incubation for 15 min, the absorbance (*A*) at 530 nm was read using an EL800 microplate reader (Bio-Tek Instruments, Winooski, VT, USA). The inhibition of fructosamine formation was then determined using Equation (2):

(2) $$\%\mathrm{inhibition}=\left(1-\frac{A_{sample}-A_{blank}}{A_{control}-A_{blank}}\right)\times 100\%,$$

#### 2.4.3. BSA-MGO Assay

The antiglycative activity of the samples during the middle stage of protein glycation was assessed using a BSA-MGO assay [26]. Briefly, a reaction mixture containing 600 μL of BSA solution (50 mg/mL in 0.1 M PBS, pH 7.4), 350 μL of MGO solution (11.43 mM in water), and 50 μL of sample (extracts: 2–8 mg/mL; pure compounds: 5–20 mM in 50% methanol) was incubated at 37 °C for 7 days. Subsequently, 200 μL of each solution was transferred to a 96-well black plate, and the fluorescence of the formed AGEs was measured (*λ*_ex_ = 360 ± 20 nm, *λ*_em_ = 460 ± 20 nm). AG at final concentrations of 50–200 μg/mL or 5–20 mM was used as a positive control. The percentage inhibition of protein glycation was calculated using Equation (1) as described in Section 2.4.1, where *f_control_* is the fluorescence intensity of the MGO-containing reaction without sample, *f_sample_* is the fluorescence intensity of the MGO-containing reaction with sample, *f_blank1_* is the fluorescence intensity of a mixture containing BSA and sample but no MGO, and *f_blank2_* is the fluorescence intensity of BSA alone.

#### 2.4.4. G.K. Peptide-Ribose Assay

The antiglycative activity of the samples at the late stage of protein glycation was evaluated using G.K. peptide-ribose assay [27]. Briefly, a reaction mixture was prepared by combining 100 μL of G.K. peptide solution (40 mg/mL in 0.1 M PBS, pH 7.4), 100 μL of D-ribose solution (60 mg/mL in PBS), 50 μL of sodium azide solution (0.4 mg/mL in PBS), and 50 μL of sample (extracts: 48–1200 μg/mL; pure compounds: 0.48–12 mM in water). The mixture was incubated at 37 °C for 24 h. Subsequently, 200 μL of the solution was transferred to a 96-well black plate, and the fluorescence intensity was measured at *λ*_ex_ = 360 ± 20 nm and *λ*_em_ = 460 ± 20 nm. AG at final concentrations of 48–1200 μg/mL or 0.48–12 mM was used as the positive control. The percentage inhibition of protein glycation was calculated using Equation (1) as described in Section 2.4.1, where *f_control_* is the fluorescence intensity of the mixture of G.K. peptide and ribose without sample; *f_sample_* is the fluorescence intensity of the mixture of G.K. peptide and ribose with sample; *f_blank_*_1_ is the fluorescence intensity of the mixture containing G.K. peptide and sample, but without D-ribose; *f_blank_*_2_ is the fluorescence intensity of G.K. peptide alone.

#### 2.4.5. MGO Scavenging Assay

The MGO scavenging capacity was determined by quantifying residual MGO post-reaction. A test mixture, composed of 250 μL of PBS (0.1 M, pH 7.4), 50 μL of MGO (2 mM in PBS), and 50 μL of sample (0.5–2.0 mM in 50% methanol) or 50% methanol (blank group), was incubated for 1 h at 37 °C. The reaction was quenched with 2 μL of acetic acid. The remaining MGO was then derivatized by adding 50 μL of 40 mM 1,2-diaminobenzene (in water), followed by a 30 min incubation at 37 °C to form 2-methylquinoxaline, which was quantified via HPLC (injection volume 15 μL) with detection at 315 nm [28]. AG was employed as the positive control. The MGO scavenging activity was then determined using Equation (3):

(3) $$\%\mathrm{inhibition}=\left(1-\frac{A_{sample}}{A_{blank}}\right)\times 100\%$$

where *A_sample_* is the HPLC peak area of 2-methylquinoxaline in the sample group, and *A_blank_* is the corresponding peak area in the blank group.

#### 2.4.6. Measurement of MGO-Astilbin Adducts

For the preparation of MGO-astilbin adducts, a reaction mixture containing 350 μL of PBS (0.1 M, pH 7.4), 25 μL of astilbin (4 mM in 50% methanol), and 25 μL of MGO (0, 4, 20, or 40 mM in PBS) was incubated for 1 h at 37 °C. The reaction was quenched with 2 μL of acetic acid. The resulting MGO-astilbin adducts were subsequently analyzed by HPLC (injection volume 5 μL) and characterized using LC-ESI-MS on an AB Sciex QTrap^®^ 4500 system (Sciex, Foster City, CA, USA) operated in negative ion mode (injection volume 5 μL).

### 2.5. Screening of Antiglycative Compounds by MGO-HPLC Assay

To identify potential MGO scavengers in the 80% MeOH extract, a solution of the extract (1 mg/mL) was incubated with MGO (400 μg/mL) in PBS (0.1 M, pH 7.4) for 1 h at 37 °C. The reaction was quenched by adding glacial acetic acid (0.2% *v*/*v*), and the solution was analyzed by HPLC (injection volume 10 μL). Compounds exhibiting a decrease in HPLC peak area in the MGO-treated group, compared to an MGO-free control, were considered potential scavengers. To account for possible pH-induced structural changes in certain compounds in PBS, a separate control was performed by incubating the extract in water under otherwise identical conditions.

### 2.6. Partition of the 80% MeOH Extract

Partitioning of the 80% MeOH extract (38.56 g) was performed with EtOAc/MeOH/water (6:1:5, *v*/*v*; 4.8 L). After 48 h of settling, the upper phase was collected. The partitioning was repeated once by adding 2.4 L of EtOAc to the lower phase. The combined upper phases were evaporated to dryness (37 °C), yielding the partitioned upper layer (PU, 13.48 g). The lower phase was separately evaporated to obtain the partitioned lower layer (PL, 25 g).

### 2.7. Precipitation and Purification of the Major Compound

#### 2.7.1. Precipitation of the Major Compound

The PU sample (4.8 g) was dissolved in 40% aqueous methanol (75 mL) with stirring and then concentrated to approximately 25 mL by heating at 60 °C to precipitate the major compound. After settling at 24 °C, the mixture was separated into the supernatant (PUS) and precipitate (PUP), which were dried to obtain 1.21 g and 3.58 g of the respective fractions.

#### 2.7.2. Purification of the Major Compound by Pre-HPLC

Purification of the major compound was achieved using a JAI LC-908 preparative HPLC system with a JAIGEL-GS310 column (Φ20 × 500 mm). A sample of PUP (126 mg in 2 mL of 80% methanol) was injected and eluted at 4 mL/min with a stepwise gradient of 612 mL of 40% methanol and 600 mL of 50% methanol (each containing 0.1% TFA), affording 33.1 mg of the target compound.

### 2.8. Fractionation of PUS by Column Chromatography

The PUS sample (1.23 g), after dissolution in 3.5 mL of 50% methanol and 0.45 μm filtration, was fractionated by Sephadex LH-20 column chromatography (SCC, Φ24 × 600 mm) using 50% methanol as the eluent (0.735 mL/min). Following an initial 120 mL elution volume, fractions were collected automatically every 6 min. HPLC analysis guided the combination of fractions into three pooled samples: SCC 123–127 (53.2 mg, compounds **2**/**8**), SCC 135–150 (232.9 mg, compounds **1**/**6**/**8**), and SCC 173–206 (101.5 mg, compounds **6**/**9**/**11**).

### 2.9. HSCCC Separation

#### 2.9.1. HSCCC Instrumentation

A TBE 300C system (Tauto Biotech., Shanghai, China) was employed for all HSCCC separations. The apparatus featured a three-coil configuration (2.6 mm I.D., 300 mL total volume). The separation unit was integrated with an Isolera FLASH system (Biotage, Uppsala, Sweden) that provided the pumping, UV detection (at 280 nm), and automated fraction collection functionalities.

#### 2.9.2. Screening and Preparation of HSCCC Solvent System

The selection of solvent systems for HSCCC was guided by the partition coefficients (*K*) of the target compounds. Several biphasic systems were prepared by vigorously mixing equal volumes of MeOH/water (1:5, *v*/*v*) with each of the following organic phases: *n*-hexane/EtOAc (1.5:5, *v*/*v*), *n*-hexane/EtOAc (1:5, *v*/*v*), EtOAc, and EtOAc/*n*-BuOH (6:1, *v*/*v*) [29]. The 80% MeOH extract (~1 mg) was dissolved and partitioned in 1.5 mL tubes using equal volumes (500 μL each) of upper and lower phases from candidate biphasic systems. After phase separation, 200 μL aliquots from each layer were evaporated by EZ-2 Plus centrifugal evaporator (Genevac Ltd., Vally Center, NY, USA) at 37 °C. The residues were redissolved in 200 μL of 80% methanol and analyzed by HPLC (injection volume 10 μL). The *K* value for each compound was determined as the ratio of HPLC peak areas between the upper and lower phases (*K* = A_upper_/A_lower_). Solvent systems were considered suitable when target compounds exhibited *K* values ranging from 0.5 to 2.0 [30]. Based on this criterion, three systems were selected: (*n*-hexane/EtOAc 1:5, *v*/*v*)/(MeOH/water 1:5, *v*/*v*) (1:1) for separating compounds **1**, **6**, and **8** from SCC 135–150; EtOAc/(MeOH/water 1:5, *v*/*v*) (1:1) for separating **2** and **8** from SCC 123–127; and (*n*-hexane/EtOAc 1.5:5, *v*/*v*)/(MeOH/water 1:5, *v*/*v*) (1:1) for separating **9** and **11** from SCC 173–206. Each selected solvent system was equilibrated in a separatory funnel, and the separated phases were degassed via sonication for 30 min prior to use.

#### 2.9.3. Separation of Compounds **1**, **6** and **8** by HSCCC

The HSCCC coil was first loaded with the upper phase of the solvent system (*n*-hexane/EtOAc 1:5, *v*/*v*)/(MeOH/water 1:5, *v*/*v*) (1:1, *v*/*v*) as the stationary phase. Following this, the apparatus was roated at 870 rpm while the mobile phase (lower layer) was pumped through at 3 mL/min to establish hydrodynamic equilibrium. A sample of SCC fractions 135–150 (232.9 mg), dissolved in 20 mL of a 1:1 (*v*/*v*) mixture of the mobile and stationary phases, was injected. Elution was continued with the mobile phase at 3 mL/min. After compound **1** was eluted at approximately 170 min, the system was switched to recycling mode by directing the effluent from the UV detector back into the column inlet (Figure S1). Compounds **8** and **6** were successfully separated after three recycling cycles. The final retention of the stationary phase was 66.7% (200 mL of the 300 mL column volume).

#### 2.9.4. Separation of Compounds **2** and **8** by HSCCC

The separation of compounds **2** and **8** was performed using the lower phase of the EtOAc/(MeOH/water 1:5, *v*/*v*) (1:1, *v*/*v*) system as the stationary phase. After equilibration at 886 rpm with the upper phase as the mobile phase (3 mL/min), a sample of SCC 123–127 (52 mg in 10 mL of a 1:1 phase mixture) was injected. The process achieved a stationary phase retention of 73%.

#### 2.9.5. Separation of Compounds **9** and **11** by HSCCC

The separation of compounds **9** and **11** was initiated using conventional HSCCC using the solvent system (*n*-hexane/EtOAc 1.5:5, *v*/*v*)/(MeOH/water 1:5, *v*/*v*) (1:1, *v*/*v*), employing the lower and upper phases as the stationary and mobile phases, respectively. After loading the stationary phase into the column, the system was operated at 880 rpm with mobile phase elution at 3 mL/min until equilibrium was achieved. Injection of 100 mg of SCC fractions 173–206 (dissolved in 16 mL of a 1:1 phase mixture) yielded a mixture containing compounds **9** and **11**.

Subsequently, this mixture (33 mg) was subjected to recycling HSCCC for further purification using the same solvent system. The sample was dissolved in 12 mL of a 1:1 phase mixture and injected. The eluate was recycled from the detector outlet back to the column inlet. After four complete cycles, the eluate was collected. The stationary phase retention values were 75% for the conventional run and 79% for the recycling run.

### 2.10. Structure Identification

Structure identification of the separated compounds was achieved by analysis of NMR data (600 MHz, Bruker Avance Neo 600 Ultra ShieldTM, Bruker Corporation, Ettlingen, German) and MS data (ESI-negative mode; AB Sciex QTrap^®^ 5500, Foster City, CA, USA), with confirmation by comparison to literature data.

Notably, a workflow figure (Figure S3) was added to enhance the clarity of the methodological strategy for the screening, isolation, and identification of antiglycative compounds from Pasuchaca.

### 2.11. Quantification of Astilbin in the Extracts and Partitions of Pasuchaca

The quantification of astilbin was performed by HPLC with an injection volume of 10 μL. A stock solution of astilbin (1.00 mg/mL in 50% methanol) was serially diluted with the same solvent to generate a calibration curve over the concentration range of 2.5–100 μg/mL (*x*-axis: concentration; *y*-axis: HPLC peak area; *n* = 3 per concentration). The method was validated by determining the limit of detection (LOD) and limit of quantification (LOQ) at signal-to-noise ratios of 3 and 10, respectively. The precision of the method, expressed as the relative standard deviation (RSD) of the peak areas, was evaluated using astilbin solutions at 25 and 70 μg/mL (*n* = 6 for each concentration).

### 2.12. Statistical Analysis

The half-maximal inhibitory concentrations (IC_50_ values) for anti-glycation agents were determined using either logarithmic analysis or linear regression, depending on which method yielded a higher regression coefficient. SPSS version 25 software (IBM, New York, NY, USA) was used to conduct statistical analyses of the activity data using one-way analysis of variance (ANOVA) followed by Tukey’s multiple comparison test.

## 3. Results

### 3.1. Antiglycative Activity and HPLC Profiles of the Extracts of Pasuchaca

**Figure 1:**
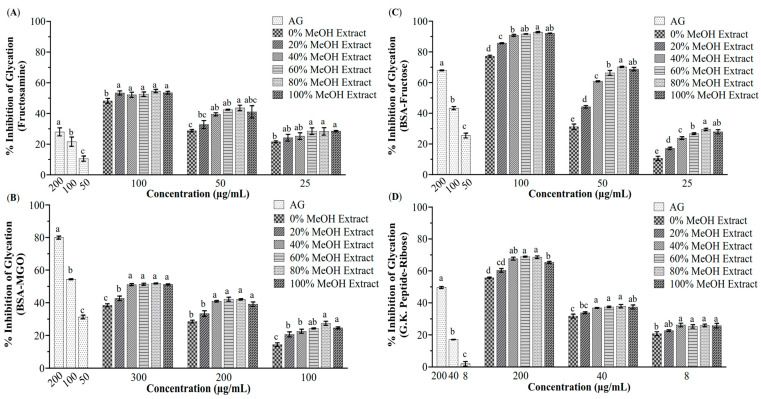
Antiglycation activities of the extracts of Pasuchaca against the formation of fructosamine-mediated early-stage AGEs (**A**), BSA-MGO mediated middle-stage AGEs (**B**), BSA-fructose mediated whole-stage AGEs (**C**), and G.K. peptide-ribose mediated late-stage AGEs (**D**). Bars marked with different letters were significantly different from each other (*p* < 0.05). Aminoguanidine hydrochloride (AG) was used as a positive control.

All the 0–100% MeOH extracts of Pasuchaca showed anti-glycative activities, among which the 80% MeOH extract exhibited the highest (Figure 1). In particular, the IC_50_ values of the 80% MeOH extract against the formation of fructosamine-mediated early-stage AGEs, BSA-MGO mediated middle-stage AGEs, BSA-fructose mediated whole-stage AGEs, and G.K. peptide-ribose mediated late-stage AGEs were 75.21 μg/mL, 277.57 μg/mL, 36.49 μg/mL, and 109.87 μg/mL, respectively; whereas the inhibition rate of the positive control, AG, on the formation of fructosamine-mediated early-stage AGEs at 100 μg/mL was 21.61%, and the IC_50_ values of AG against the formation of BSA-MGO mediated middle-stage AGEs, BSA-fructose mediated whole-stage AGEs, and G.K. peptide-ribose mediated late-stage AGEs were 86.33 μg/mL, 113.95 μg/mL, and 230.95 μg/mL, respectively. The dose–response data used for the IC_50_ calculations were in Table S1.

It is noteworthy that all 0–100% MeOH extracts had a major constituent (Figure S2), suggesting that the major compound may be the key contributor to the antiglycative activity of the extracts.

### 3.2. Screening of Antiglycative Compounds Using MGO-HPLC Assay

**Figure 2:**
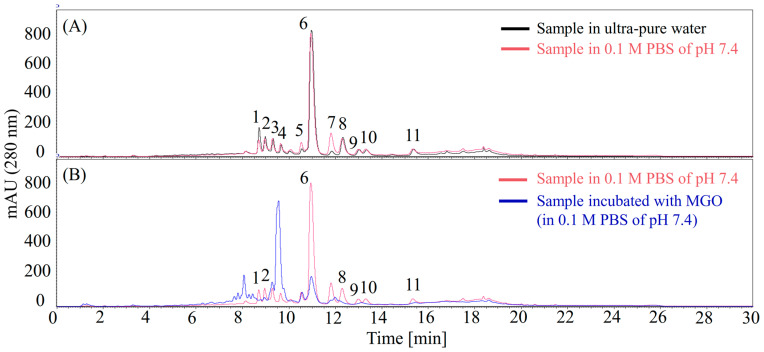
Screening of methylglyoxal (MGO) scavengers in the 80% MeOH extract of Pasuchaca by MGO-HPLC assay. (**A**) Incubation of the sample in water (black line) and 0.1 M PBS at pH 7.4 (pink line) for 1 h. (**B**) Incubation of the sample with MGO (blue line) or without MGO (pink line) in 0.1 M PBS at pH 7.4 for 1 h. Compounds **1**, **2**, **6**, **8**, **9**, **10**, and **11** were screened as potential MGO scavengers.

Upon reaction with MGO in 0.1 M PBS (pH 7.4), eight compounds showed a notable depletion in their HPLC peak areas when compared to the control group without MGO (Figure 2B). A parallel experiment was conducted by incubating the sample in water to assess the potential effects of 0.1 M PBS buffer at pH 7.4 on the compounds. Peak **7** was apparently an artificial HPLC peak produced via incubation in 0.1 M PBS buffer at pH 7.4 and was therefore excluded as target compound (Figure 2A). Finally, compounds **1**, **2**, **6**, **8**, **9**, **10**, and **11** were finally screened as potential MGO scavengers.

### 3.3. Partition and Antiglycative Activity of the Partitioned Upper and Lower Layers of the 80% MeOH Extract

The solvent system screening identified EtOAc/MeOH/water (6:1:5, *v*/*v*), the same as EtOAc/(MeOH/Water 1:5, *v*/*v*) (1:1, *v*/*v*), as optimal based on partition coefficients which provided *K* > 2 for most of the target compounds while yielding *K* = 0.1 for compound **4**, ensuring effective separation, with the targets concentrating in the upper phase while compound **4** remained in the lower phase (Table 1).

**Figure 3:**
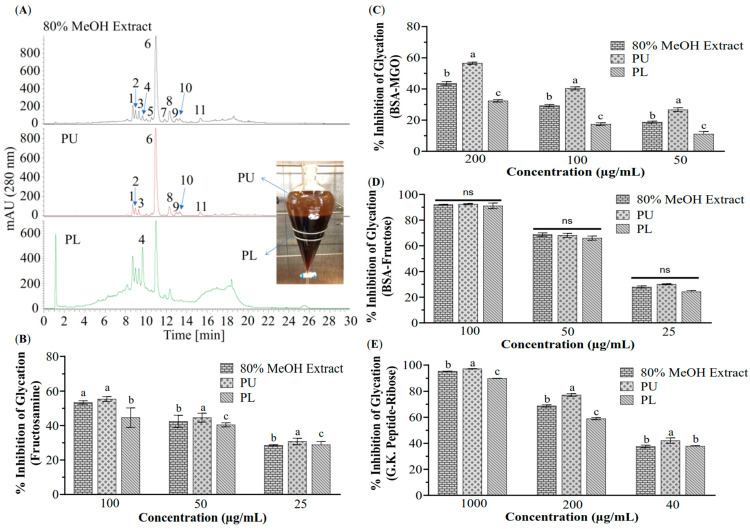
Partition of the 80% MeOH extract of Pasuchaca and antiglycative activity of the partitions. (**A**) HPLC profiles of the partitioned upper layer (PU) and lower layer (PL) of the 80% MeOH extract. Antiglycative activity of the 80% MeOH extract and its PU and PL against the formations of fructosamine-mediated early-stage AGEs (**B**), MGO-BSA mediated middle-stage AGEs (**C**), BSA-fructose mediated whole-stage AGEs (**D**), and G.K. peptide-ribose mediated late-stage AGEs (**E**). Bars marked with different letters were significantly different from each other (*p* < 0.05). ns stands for not significant (*p* > 0.05).

Finally, 13.48 g of PU and about 25 g of PL were obtained and the target compounds and the non-target compound **4** were well separated (Figure 3A). Moreover, as shown in Figure 3B–E, the PU sample exhibited higher antiglycative activity than PL and the 80% MeOH extract against the formations of fructosamine-mediated early-stage AGEs (80% MeOH extract, IC_50_ 82.05 μg/mL; PU, IC_50_ 71.51 μg/mL; PL, 45.93% inhibition at 100 μg/mL), BSA-MGO mediated middle-stage AGEs (80% MeOH extract, 43.55% inhibition at 200 μg/mL; PU, IC_50_ 161.14 μg/mL; PL, 32.43% inhibition at 200 μg/mL), BSA-fructose mediated whole-stage AGEs (80% MeOH extract, IC_50_ 38.08 μg/mL; PU, IC_50_ 37.19 μg/mL; PL, IC_50_ 40.45 μg/mL), and G.K. peptide-ribose mediated late-stage AGEs (80% MeOH extract, IC_50_ 76.80 μg/mL; PU, IC_50_ 58.68 μg/mL; PL, IC_50_ 96.30 μg/mL). The dose–response data used for the IC_50_ calculations were in Table S2.

### 3.4. Separation of the Major Compound and Fractionation of the Minor Compounds

**Figure 4:**
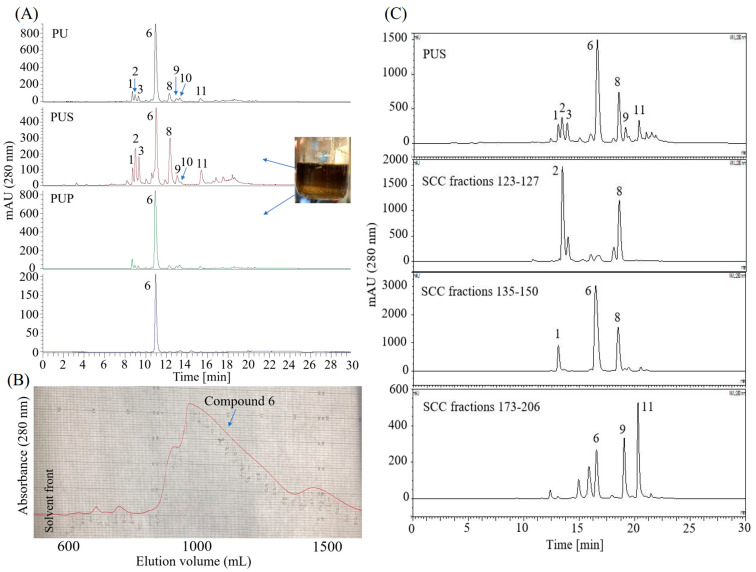
HPLC and pre-HPLC chromatograms of the precipitation and separation process of the 80% MeOH extract. (**A**) The supernatant (PUS) and precipitate (PUP) of the partitioned upper layer (PU) of the 80% MeOH extract and the purified compound **6** by pre-HPLC; (**B**) separation of compound **6** by Pre-HPLC; (**C**) fractionation of the target compounds by sephadex LH-20 column chromatography (SCC).

Through solvent evaporation, the major compound **6** in PU was precipitated to enrich the minor compounds **1**, **2**, **6**, **8**–**11** and facilitate subsequent separation, as outlined in Section 2.7.1. Starting from 4.8 g of PU, this process yielded 3.58 g of the precipitate fraction (PUP) and 1.21 g of the supernatant fraction (PUS). The minor compounds were effectively concentrated into PUS, while PUP consisted predominantly of the target compound **6** (Figure 4A). Next, 126 mg of PUP was subjected to pre-HPLC purification, yielding 33.1 mg of compound **6** (Figure 4A,B). Furthermore, 1.23 g of PUS was separated by Sephadex LH-20 column chromatography (SCC), yielding several fractions enriched with target compounds: 53.2 mg of SCC fractions 123–127 (containing **2** and **8**), 232.9 mg of SCC fractions 135–150 (containing **1**, **6**, and **8**), and 101.5 mg of SCC fractions 173–206 (primarily containing **6**, **9**, and **11**) (Figure 4C).

### 3.5. Separation of the Target Compounds Using HSCCC

**Figure 5:**
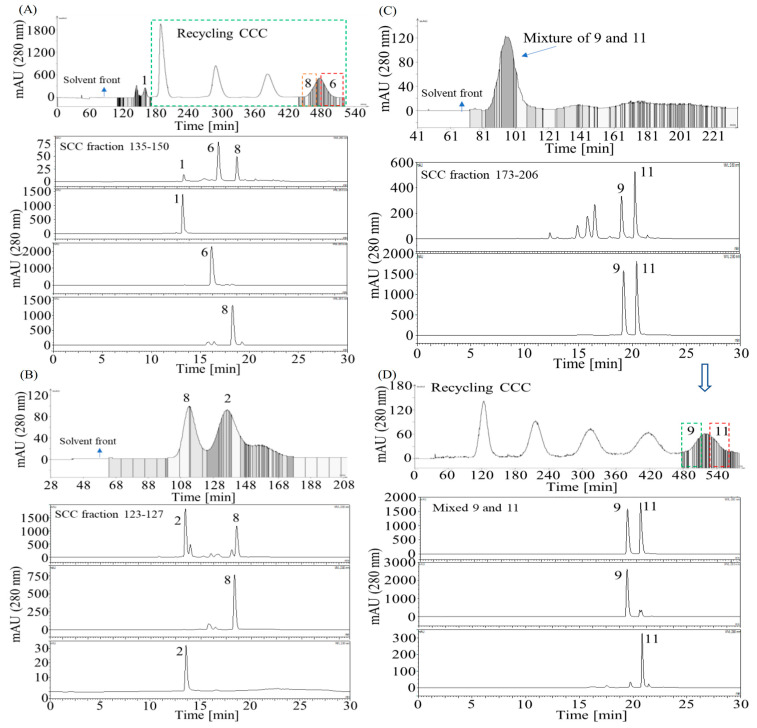
Separation of the target compounds from the Sephadex LH-20 column chromatography (SCC) fractions of Pasuchaca by conventional HSCCC and recycling HSCCC. (**A**) Separation of compounds **1**, **6**, and **8** from SCC fractions 135–150 by HSCCC and recycling HSCCC (171–540 min, marked by a green box) using the solvent system (*n*-hexane/EtOAc 1:5, *v*/*v*)/(MeOH/water 1:5, *v*/*v*) (1:1, *v*/*v*), whereas the chromatographic peak positions of compounds **6** and **8** in recycling HSCCC were marked by red boxes; (**B**) Separation of compounds **2** and **8** from SCC fractions 123–127 by conventional HSCCC using the solvent system (EtOAc/(MeOH/Water 1:5, *v*/*v*) (1:1, *v*/*v*); (**C**) Separation of the mixture of **9** and **11** from SCC fractions 173–206 by conventional HSCCC using the solvent system (*n*-hexane/EtOAc 1.5:5, *v*/*v*)/(MeOH/water 1:5, *v*/*v*) (1:1, *v*/*v*); (**D**) Separation of compounds **9** and **11** from the mixture of **9** and **11** by recycling HSCC using the solvent system (*n*-hexane/EtOAc 1.5:5, *v*/*v*)/(MeOH/water 1:5, *v*/*v*) (1:1, *v*/*v*), whereas the chromatographic peak positions of compounds 9 and 11 in recycling HSCCC were marked by green and red boxes, respectively.

The compounds **6** and **8** could not be separated by conventional HSCCC since they share similar *K* values (Table 1). Then, a strategy to separate compound **1** (110–171 min) first by conventional HSCCC and then separate compounds **6** and **8** (171–540 min) by recycling HSCCC was proposed and conducted using the solvent system (*n*-hexane/EtOAc 1:5, *v*/*v*)/(MeOH/water 1:5, *v*/*v*) (1:1, *v*/*v*) as described in Section 2.9.3, resulting in separation of 6.3 mg of compound **1**, 37.1 mg of compound **6**, and 5.4 mg of compound **8** from 232.9 mg of SCC fractions 135–150 (Figure 5A).

The separation of compound **2** was conducted by conventional HSCCC using the solvent system (EtOAc/(MeOH/Water 1:5, *v*/*v*) (1:1, *v*/*v*) since it offered the *K* value of compound **2** with *K* = 1.42 (Table 1), yielding 11.6 mg of compound **8** and 8.0 mg of compound **2** from 52 mg of SCC fractions 123–127 (Figure 5B), as described in Section 2.9.4.

The SCC fractions 173–206 (100 mg) was first subjected to conventional HSCCC to remove the non-target compounds, as described in Section 2.9.5, yielding 35 mg of a mixture composed merely of compounds **9** and **11** (Figure 5C), which was further separated using recycling HSCCC as described in Section 2.9.5, yielding 4.6 mg of compound **9** and 10.8 mg of compound **11** (Figure 5D).

Consequently, the target compounds **1**, **2**, **6**, **7**, **8**, **9**, and **11** were separated using conventional or recycling HSCCC Figure 5). However, compound **10** was not obtained due to dispersion during fractionation by Sephadex LH-20 column chromatography.

### 3.6. Identification of the Separated Target Compounds

**Figure 6:**
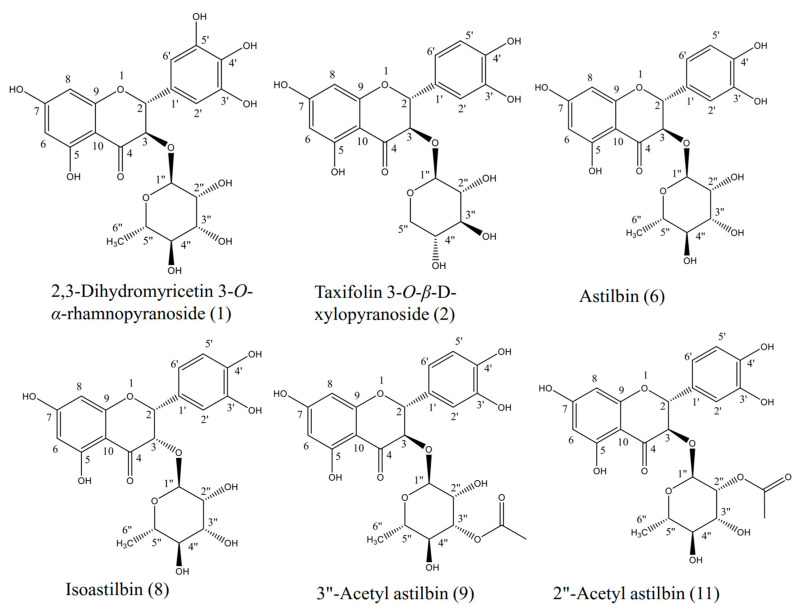
Identification of the separated compounds in Pasuchaca.

The structures of the isolated compounds are shown in Figure 6 and were identified as follows. 

2,3-Dihydromyricetin 3-*O*-*α*-rhamnopyranoside (**1**): ESI-MS/MS (negative ion) *m*/*z*, primary mass spectrum (MS) ion [M-H]^−^ 465.2; key fragment ions of the secondary mass spectrum (MS/MS) from [M-H]^−^, 447.2 [M-H_2_O-H]^−^, 339.2 [M-B ring-H]^−^, 301.0 [M-rhamnose-H_2_O-H]^−^, and 151.0. ^1^H-NMR (600 MHz, MeOD-*d*_4_) *δ*_H_: 6.53 (2H, s, H-2′, 6′ ), 5.94 (1H, d, *J* = 2.11 Hz, H-8), 5.92 (1H, d, *J* = 2.04 Hz, H-6), 5.02 (1H, d, *J* = 10.56 Hz, H-2), 4.55 (1H, d, *J* = 10.56 Hz, H-3), 4.26 (1H, m, H-5″), 4.12 (1H, brs, H-1″), 3.68 (1H, dd, *J* = 9.58, 3.29 Hz, H-3″), 3.61 (1H, dd, *J* = 3.05, 1.65 Hz, H-2″), 3.35 (1H, overlapped with solvent peak, H-4″), 1.21 (3H, d, *J* = 6.23 Hz, H-6″) [31].

(+)-Taxifolin 3-*O*-*β*-D-xylopyranoside (**2**): ESI-MS/MS (negative ion) *m*/*z*, primary MS ion [M-H]^−^ 435.3; key fragment ions of MS/MS from [M-H]^−^, 303.2 [M-xylopyranose-H]^−^, 285.2 [M-xylopyranose-H_2_O-H]^−^, and 151.1. ^1^H-NMR (600 MHz, MeOD-*d*_4_) *δ*_H_: 6.86 (1H, d, *J* = 2.02 Hz, H-2′), 6.74 (1H, dd, *J* = 8.22, 2.00 Hz, H-6′), 6.70 (1H, d, *J* = 8.12 Hz, H-5′), 5.82 (1H, d, *J* = 2.14 Hz, H-8), 5.81 (1H, d, *J* = 2.12 Hz, H-6), 3.85 (1H, dd, *J* = 10.77, 4.69 Hz, H-5″a), 3.79 (1H, d, *J* = 6.05 Hz, H-1″), 3.40 (1H, m, H-4″), 3.12–3.17(2H, m, H-2″, H-3″), 2.98 (1H, dd, *J* = 11.78, 8.46 Hz, H-5″b) [32].

Astilbin (**6**): ESI-MS/MS (negative ion) *m*/*z*, primary MS ion [M-H]^−^ 449.2; key fragment ions of MS/MS from [M-H]^−^, 303.1 [M-rhamnose-H]^−^, 285.2 [M- rhamnose-H_2_O-H]^−^, and 151.1 [33]. ^1^H-NMR (600 MHz, MeOD-*d*_4_) *δ*_H_: 6.97 (1H, d, *J* = 1.96 Hz, H-2′), 6.86 (1H, dd, *J* = 8.19, 2.03 Hz, H-6′), 6.83 (1H, d, *J* = 8.12 Hz, H-5′), 5.94 (1H, d, *J* = 2.14 Hz, H-8), 5.92 (1H, d, *J* = 2.15 Hz, H-6), 5.09 (1H, d, *J* = 10.74 Hz, H-2), 4.60 (1H, d, *J* = 10.73 Hz, H-3), 4.27 (1H, m, H-5″), 4.07 (1H, d, *J* = 1.47 Hz, H-1″), 3.68 (1H, dd, *J* = 9.57, 3.30 Hz, H-3″), 3.56 (1H, dd, *J* = 3.26, 1.71 Hz, H-2″), 3.33 (1H, overlapped with solvent peak, H-4″), 1.21 (3H, d, *J* = 6.24 Hz, H-6″). ^13^C-NMR (150 MHz, MeOD-d4) *δ*_C_: 194.60 (C-4), 167.21 (C-7), 164.12 (C-5), 162.71 (C-9), 145.98 (C-4′), 145.14 (C-3′), 127.78 (C-1′), 119.08 (C-6′), 114.91 (C-5′), 114.08 (C-2′), 101.09 (C-10), 100.75 (C-1″), 95.97 (C-8), 94.85 (C-6), 82.56 (C-3), 77.16 (C-2), 72.40 (C-4″), 70.76 (C-3″), 70.38 (C-2″), 69.11 (C-5″), 16.45 (C-6″) [34,35].

Isoastilbin (**8**): ESI-MS/MS (negative ion) *m*/*z*, primary MS ion [M-H]^−^ 449.2; key fragment ions of MS/MS from [M-H]^−^, 303.1 [M-rhamnose-H]^−^, 285.0 [M- rhamnose-H_2_O-H]-, and 151.0. ^1^H-NMR (600 MHz, MeOD-*d*_4_) *δ*_H_: 6.96 (1H, d, *J* = 1.69 Hz, H-2′), 6.86 (1H, dd, *J* = 8.25, 1.77 Hz, H-6′), 6.82 (1H, d, *J* = 8.13 Hz, H-5′), 5.99 (1H, d, *J* = 1.52 Hz, H-8), 5.94 (1H, d, *J* = 1.98 Hz, H-6), 5.44 (1H, d, *J* = 2.22 Hz, H-2), 4.98 (1H, brs, H-1″), 3.69 (1H, dd, *J* = 3.17, 1.51 Hz, H-2″), 3.47 (1H, dd, *J* = 9.59, 3.35 Hz, H-3″), 3.22 (1H, t, *J* = 9.56 Hz, H-4″), 2.49 (1H, td, *J* = 9.48, 6.11 Hz, H-5″), 0.96 (3H, d, *J* = 6.21 Hz, H-6″) [36].

3″-Acetyl astilbin (**9**): ESI-MS/MS (negative ion) *m*/*z*, primary MS ion [M-H]^−^ 491.2; key fragment ions of MS/MS from [M-H]^−^, 303.2 [M-acetyl rhamnose-H]^−^, 285.2 [M-acetyl rhamnose-H_2_O-H]^−^, and 151.0. ^1^H-NMR (600 MHz, MeOD-*d*_4_) *δ*_H_: 6.99 (1H, d, *J* = 1.69 Hz, H-2′), 6.87 (1H, dd, *J* = 8.11, 1.72 Hz, H-6′), 6.82 (1H, d, *J* = 8.08 Hz, H-5′), 5.94 (1H, brs, H-8), 5.92 (1H, brs, H-6), 5.11 (1H, d, *J* = 10.37 Hz, H-2), 4.95 (1H, dd, *J* = 9.95, 2.11 Hz, H-3″), 4.60 (1H, d, *J* = 10.92 Hz, H-3), 4.43 (1H, m, H-5″), 4.01 (1H, d, *J* = 0.88 Hz, H-1″), 3.70 (1H, dd, *J* = 2.77, 1.90 Hz, H-2″), 2.10 (3H, s, -CH_3_), 1.23 (3H, d, *J* = 6.22 Hz, H-6″) [37].

2″-Acetyl astilbin (**11**): ESI-MS/MS (negative ion) *m*/*z*, primary MS ion [M-H]^−^ 491.2; key fragment ions of MS/MS from [M-H]^−^, 303.0 [M-acetyl rhamnose-H]^−^, 285.0 [M-acetyl rhamnose-H_2_O-H]^−^, and 151.0. ^1^H-NMR (600 MHz, MeOD-*d*_4_) *δ*_H_: 6.96 (1H, d, *J* = 1.29 Hz, H-2′), 6.80 (1H, brs, H-6′), 6.79 (1H, brs, H-5′), 5.94 (1H, d, *J* = 1.93 Hz, H-8), 5.93 (1H, brs, H-6), 5.13 (1H, d, *J* = 10.37 Hz, H-2), 4.95 (1H, dd, *J* = 3.63, 1.87 Hz, H-2″), 4.56 (1H, d, *J* = 10.32 Hz, H-3), 4.21 (1H, m, H-5″), 3.96 (1H, brs, H-1″), 3.84 (1H, dd, *J* = 9.61, 3.54 Hz, H-3″), 3.27 (1H, t, *J* = 9.63 Hz, H-4″), 2.00 (3H, s, -CH_3_), 1.20 (3H, d, *J* = 6.22 Hz, H-6″) [37].

### 3.7. Antiglycative Activity of the Major Compound Astilbin

**Figure 7:**
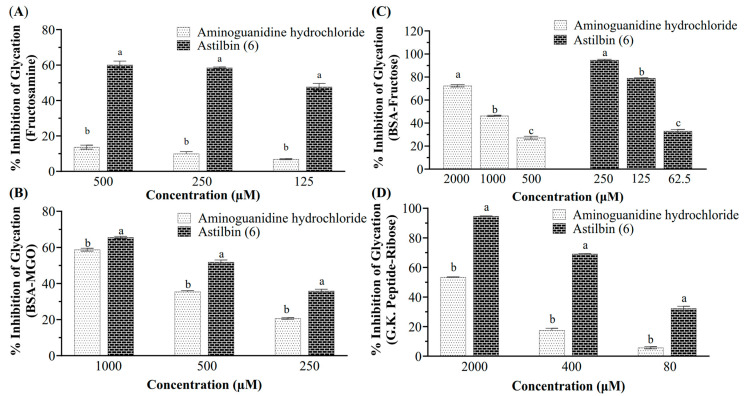
Antiglycative activity of astilbin separated from Pasuchaca. Inhibitory activities of astilbin against the formation of fructosamine-mediated early-stage AGEs (**A**), BSA-MGO mediated middle-stage AGEs (**B**), BSA-fructose mediated whole-stage AGEs (**C**), and G.K. peptide-ribose mediated late stage AGEs (**D**). Bars marked with different letters were significantly different from each other (*p* < 0.05). Aminoguanidine hydrochloride was used as a positive control.

Astilbin exhibited significantly higher antiglycative activity than the positive control AG against the formations of fructosamine-mediated early-stage AGEs (astilbin, IC_50_ 149.52 μM; AG, 13.69% inhibition at 500 μM), BSA-MGO mediated middle-stage AGEs (astilbin, IC_50_ 475.45 μM; AG, IC_50_ 816.27 μM), BSA-fructose mediated whole-stage AGEs (astilbin, IC_50_ 84.29 μM; AG, IC_50_ 1042.56 μM), and G.K. peptide-ribose mediated late-stage AGEs (astilbin, IC_50_ 182.60 μM; AG, IC_50_ 1833.63 μM), as shown in Figure 7. The dose–response data used for the IC_50_ calculations were in Table S3.

### 3.8. MGO Scavenging Activity of the Target Compounds

**Figure 8:**
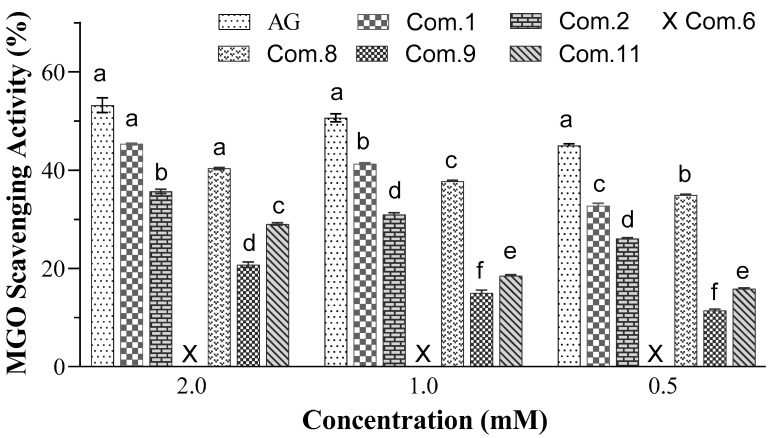
Methylglyoxal scavenging activity of compounds **1**, **2**, **6**, **8**, **9**, and **11** from Pasuchaca. The compounds are 2,3-dihydromyricetin 3-*O*-α-rhamnopyranoside (Com.1), (+)-taxifolin 3-*O*-*β*-D-xylopyranoside (Com.2), astilbin (Com.6), isoastilbin (Com.8), 3″-acetyl astilbin (Com.9), and 2″-acetyl astilbin (Com.11). Aminoguanidine hydrochloride (AG) was used as a positive control. The methylglyoxal (MGO) scavenging activity of astilbin (Com.6) was not measurable since the HPLC peaks of 2-methyl quinoxaline and the produced MGO-astilbin adducts overlapped. Different letters indicate significant differences in the compounds’ activity in scavenging methylglyoxal at the same concentration (*p* < 0.05).

The target compounds 2,3-dihydromyricetin 3-*O*-α-rhamnopyranoside (**1**), (+)-taxifolin 3-*O*-*β*-D-xylopyranoside (**2**), isoastilbin (**8**), 3″-acetyl astilbin (**9**), and 2″-acetyl astilbin (**11**) all exhibited MGO scavenging activity, although lower than the positive control AG, validating the application of MGO-HPLC assay to screen MGO scavengers from natural products (Figure 8). However, the MGO scavenging activity of astilbin could not be measured as the HPLC peaks of 2-methyl quinoxaline and the produced MGO-astilbin adducts overlapped.

### 3.9. Identification of Astilbin-MGO Adducts by LC-MS

**Figure 9:**
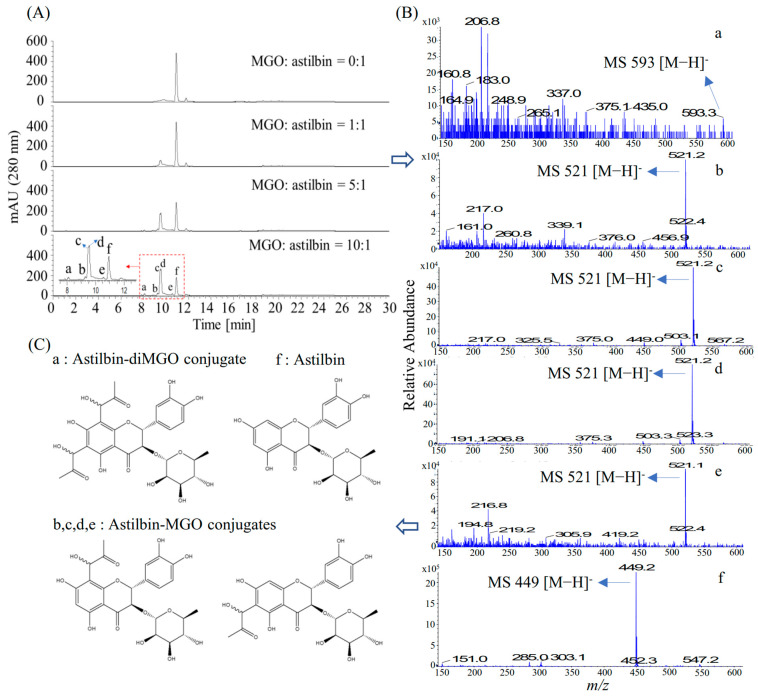
Identification of methyl glyoxal (MGO)-astilbin adducts by LC-ESI-MS. (**A**) Chromatograms of samples after incubating astilbin with different concentrations of MGO for 1 h at 37 °C; (**B**) Identification of compounds a–f by LC-MS; (**C**) The structures of compounds a–f.

The astilbin-MGO adducts were identified using LC-ESI-MS after incubation of the mixture of MGO and astilbin for 1 h (37 °C) at four ratios (0:1, 1:1, 5:1, and 10:1) (Figure 9A). Two major peaks (c and d) appeared after incubation of MGO and astiblin (peak f), which were identified as astilbin-MGO conjugates by showing molecular ions of *m*/*z* 521 [M−H]^−^ (Figure 9B). Moreover, three minor peaks (a, b, and e) appeared at high ratios of MGO to astilbin (5:1 and 10:1) (Figure 9A). Peaks b and e were identified as astilbin-MGO conjugates by showing molecular ions of *m*/*z* 521 [M−H]^−^ (Figure 9B), while peak a was identified as a astilbin-diMGO conjugate by showing a molecular ion of *m*/*z* 593.3 [M−H]^−^ (Figure 9B). The structures of the astilbin-MGO conjugates and astilbin-diMGO conjugate are shown in Figure 9C. These results validate that astilbin is an MGO scavenger.

### 3.10. Quantification of Astilbin

The HPLC method for quantification of astilbin was validated for linearity, sensitivity, and precision. A standard curve constructed over the range 2.5–100 μg/mL (injection volume 10 μL) demonstrated good linearity (r^2^ = 0.9973)., The limits of detection (LOD) and quantification (LOQ) of astilbin were determined to be 0.625 and 2.00 μg/mL, respectively (Table S4). Method precision, expressed as relative standard deviation (RSD) was satisfactory: intra-day RSDs were 5.53% and 3.59% at 25.00 and 70.00 μg/mL, respectively, while inter-day RSDs were 5.97% and 5.51% at the same concentrations.

As shown in Table 2, the astilbin is rich in the 40–100% extracts of Pasuchaca (171.47–252.41 mg/g), among which the 80% MeOH extract has the highest content of astilbin (252.41 mg/g). In particular, the astilbin content in the partitioned upper layer of the 80% MeOH extract was up to 541.04 mg/g. Furthermore, the calculated astilbin content in the dried raw aerial parts was 86.68 mg/g.

## 4. Discussion

The present study identified Pasuchaca as a rich source of antiglycative compounds, with the flavonoid astilbin being the principal active constituent. Our findings provide a scientific basis for the traditional use of Pasuchaca in managing diabetes and related complications.

The initial screening of different methanol extracts revealed that the 80% MeOH extract possessed the highest antiglycative activity, effectively inhibiting AGE formation at the early, middle, late, and whole stages of the glycation process (Figure 1). The HPLC profiles consistently showed a dominant peak across all active extracts (Figure S1), suggesting a major contributor to the bioactivity. The subsequent application of the MGO-HPLC assay proved to be an efficient strategy for rapidly pinpointing potential MGO scavengers within the complex plant matrix (Figure 2). This method successfully identified seven compounds whose peaks diminished upon reaction with MGO, indicating their ability to trap this key glycation precursor.

The removal of compound **4** via solvent-solvent partitioning was critical for successful isolation, as its high viscosity and low solubility had previously impeded the separation of the 80% MeOH extract by both HSCCC and column chromatography in our pre-tests. Having established that the EtOAc/MeOH/Water (6:1:5, *v*/*v*) system, equivalent to EtOAc/(MeOH/Water 1:5, *v*/*v*) (1:1, *v*/*v*), was suitable for partitioning astilbin and its derivatives (Table 1, Figure 3A), an integrated purification strategy was implemented. This combined solvent partitioning, precipitation, Sephadex LH-20 chromatography, and HSCCC, ultimately enabling the isolation of the target compounds (Figure 3, Figure 4 and Figure 5). The sequential use of conventional and recycling HSCCC modes was essential for separating compounds with highly similar partition coefficients, such as the pairs astilbin (**6**)/isoastilbin (**8**) and 3″-acetyl astilbin (**9**)/2″-acetyl astilbin (**11**) (Table 1, Figure 5). However, the resolution of recycling HSCCC was limited, likely due to short-term mobile phase retention in the coarse-diameter vessel (Figure S1), which caused solute dispersion from the coil tube. This limitation could potentially be mitigated by employing online storage inner-recycling [38,39] and closed-loop recycling strategies [40,41], which effectively enhanced peak resolution and separation efficiency for compounds with similar *K* values by repeatedly cycling the sample through the column, thereby functionally extending the column length and reducing the detrimental effects of solute dispersion.

Structural elucidation confirmed that the isolated compounds were dihydroflavonol derivatives, with astilbin (dihydroquercetin-3-*O*-rhamnoside) identified as the most abundant. Quantification revealed that astilbin constituted over 25% of the 80% MeOH extract and more than 54% of the bioactive partitioned upper layer (PU), firmly establishing it as the key phytochemical marker for Pasuchaca. Furthermore, the astilbin content in the dried aerial parts was determined to be 86.68 mg/g (Table 2). This high level is comparable to, and often exceeds, that of other known astilbin-rich plants such as *Smilax china* L. (1.39–14.10 mg/g), *Smilax glabra* Roxb. (11.5–47.6 mg/g) and *Engelhardia roxburghiana* (20.0–86.7 mg/g) [42], underscoring Pasuchaca’s potential as a valuable source. Given astilbin’s diverse pharmacological properties, including anti-inflammatory, hypoglycemic, and antioxidant activities [43], Pasuchaca warrants further investigation for potential applications in the pharmaceutical and food industries.

A previous study reported that astilbin markedly inhibited the formation of BSA-fructose-mediated AGEs [44], our study further establishes it as a potent antiglycative agent. Astilbin exhibited significantly lower IC_50_ values than the positive control aminoguanidine across multiple glycation models, particularly in the BSA-fructose assay (astilbin, IC_50_ 84.29 μM; AG, IC_50_ 1042.56 μM) and G.K. peptide-ribose assay (astilbin, IC_50_ 182.60 μM; AG, IC_50_ 1833.63 μM) (Figure 7), demonstrating strong and broad-spectrum inhibition of AGE formation. To elucidate the mechanism, we investigated its MGO scavenging capacity. Although the direct MGO scavenging assay for astilbin was confounded by overlapping HPLC peaks (Figure 8), the LC-ESI-MS analysis provided direct evidence of the formation of mono- and di-MGO adducts with astilbin (Figure 9). This unequivocally proves that astilbin acts as an effective scavenger of MGO, thereby preventing this key precursor from modifying proteins and forming AGEs.

## 5. Conclusions

In conclusion, this study is the first to comprehensively report on the phytochemicals responsible for the antiglycative activity of Pasuchaca. By integrating a rapid MGO-HPLC screening method with efficient HSCCC separation, we successfully isolated and identified six dihydroflavonol derivatives, with astilbin being the major and most active compound. Astilbin was found in remarkably high concentrations in Pasuchaca extracts and demonstrated superior antiglycation activity compared to the positive control aminoguanidine.

These findings not only validate the traditional use of Pasuchaca but also position it as a promising, high-yield source of astilbin for the development of functional foods, nutraceuticals, and natural therapeutic agents aimed at mitigating diabetic complications and other age-related diseases associated with glycative stress. Future research should focus on in vivo studies to confirm the efficacy of astilbin from Pasuchaca and product formulation studies to develop bioavailable astilbin-rich extracts or compounds.

## 6. Patents

The technical framework presented in this study is associated with a Chinese invention patent application (Application No.: 2024104161542; Filed Data: 2 August 2024; Status: Under substantive examination by the China National Intellectual Property Administration (CNIPA); Assignee: Zhejiang University and Jinhua Institute of Zhejiang University). The patent application covers the core process described in Section 2.6, and this paper provides additional experimental validation and activity analysis of the patented scheme.

## Figures and Tables

**Table 1:** Partition coefficient (*K* value) of compounds **1**–**11** in the biphasic solvent systems.

Solvent System	*K*_upper/lower_ Values of Components 1–11										
	1	2	3	4	5	6	7	8	9	10	11
(*n*-Hex/EtOAc 2:5, *v*/*v*)/(MeOH/Water 1:5, *v*/*v*) 1:1, *v*/*v*	0.05	0.15	0.15	0.02	0.65	0.16	0.40	0.13	0.98	0.40	0.80
(*n*-Hex/EtOAc 1.5:5, *v*/*v*)/(MeOH/Water 1:5, *v*/*v*) 1:1, *v*/*v*	0.15	0.28	0.23	0.03	0.67	0.57	0.41	0.53	4.69	1.73	3.92
(*n*-Hex/EtOAc 1:5, *v*/*v*)/(MeOH/Water 1:5, *v*/*v*) 1:1, *v*/*v*	0.14	0.41	0.37	0.04	0.84	0.89	0.43	0.83	6.86	3.00	6.55
EtOAc/(MeOH/Water 1:5, *v*/*v*) 1:1, *v*/*v*	1.24	1.42	1.18	0.10	1.71	3.87	2.10	3.68	25.55	14.53	17.00
(EtOAc/*n*-BuOH 6:1, *v*/*v*)/(MeOH/Water 1:5, *v*/*v*) 1:1, *v*/*v*	2.20	2.89	2.68	0.37	4.40	4.43	2.55	3.15	22.06	9.01	16.08

Note: *n*-Hex, EtOAc, *n*-BuOH, and MeOH are the abbreviations of *n*-hexane, ethyl acetate, *n*-butanol and methanol, respectively.

**Table 2:** Contents of astilbin in the extracts and partitions of Pasuchaca.

Sample	Content (mg Compound/g Sample)
Water extract	59.73 ± 1.76
20% MeOH extract	113.34 ± 8.21
40% MeOH extract	171.47 ± 2.02
60% MeOH extract	203.97 ± 7.38
80% MeOH extract	252.41 ± 4.52
100% MeOH extract	244.33 ± 2.21
Partitioned upper layer	541.04 ± 1.02
Partitioned lower layer	20.04 ± 0.89
Dried raw material	86.68

Note: Partitioned upper and lower layers were obtained by partitioning the 80% MeOH extract of Pasuchaca using the solvent system EtOAc/MeOH/water (6:1:5, *v*/*v*). The contents of astilbin in the extracts and partitions of Pasuchaca were quantified using HPLC and were presented as mean ± standard deviations, whereas the content of astilbin in the dried raw material (aerial part) of Pasuchaca was calculated.

## Data Availability

The original contributions presented in the study are included in the article/Supplementary Materials, further inquiries can be directed to the corresponding authors.

## Supplementary Materials

The following supporting information can be downloaded at: https://www.mdpi.com/article/10.3390/foods14234167/s1, Figure S1: The plastic vessel for directing the effluent from the UV detector back into the column inlet; Figure S2: HPLC detection of the 0–100% methanol extracts of Pasuchaca; Figure S3: Schematic workflow for the screening, isolation, and identification of antiglycative compounds from Pasuchaca; Table S1: Antiglycation activities of the extracts of Pasuchaca; Table S2: Antiglycation activities of the 80% methanol extract and its partitions; Table S3: Antiglycation activities of astilbin separated from Pasuchaca; Table S4: HPLC validation data for astilbin: calibration curve, LOD, LOQ, and precision.

## Abbreviations

The following abbreviations are used in this manuscript:

AGEs	Advanced glycation end-products
MGO	Methylglyoxa
HPLC	High-performance liquid chromatography
Pre-HPLC	Preparative high-performance liquid chromatography
HSCCC	High-speed counter-current chromatography
AG	Aminoguanidine hydrochloride
TFA	trifluoroacetic acid
NBT	4-Nitro blue tetrazolium
G.K. peptide	Ac-Gly-Lys-OMe acetate salt
BSA	Bovine serum albumin
PBS	Phosphate-buffered saline
PU	Partitioned upper layer sample of the 80% methanol extract
PL	Partitioned lower layer sample of the 80% methanol extract
PUS	PU supernatant
PUP	PU precipitate
SCC	Sephadex LH-20 column chromatography
