# Supplementary material for: Pasuchaca (Geranium dielsiaum Knuth): A New Source of Astilbin with Antiglycation Activity
Source: Foods. 2025 Dec 4;14(23):4167. doi: 10.3390/foods14234167 (PMC12692292; doi:10.3390/foods14234167)
Supplement: Supplementary file 1 [file foods-14-04167-s001.zip › foods-3965984-supplementary.pdf]

Supplementary Materials

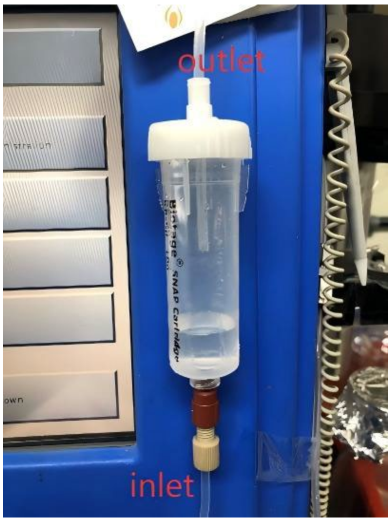

Figure S1. The plastic vessel for directing the effluent from the UV detector back into the column inlet.

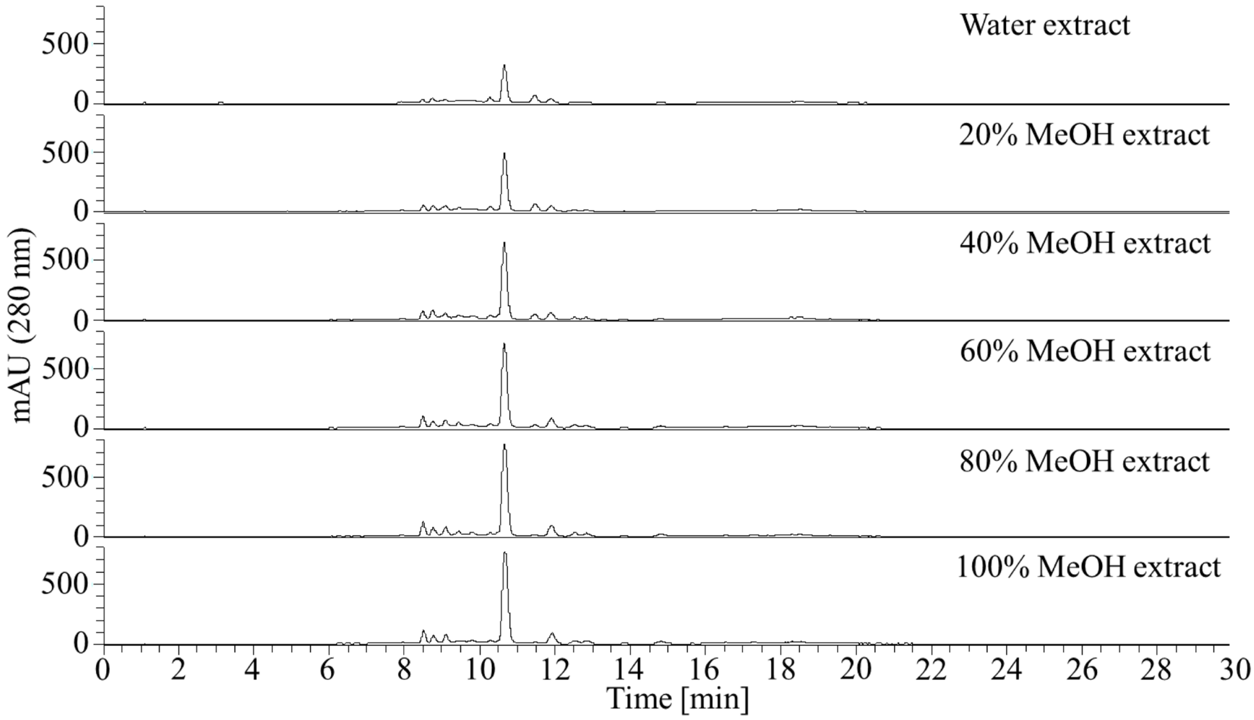

Figure S2. HPLC detection of the 0-100% methanol extracts of Pasuchaca.

Table S1. Antiglycation activities of the extracts of Pasuchaca.

Sample	Inhibition of fructosamine formation			Inhibition of BSA-MGO AGEs			Inhibition of BSA-fructose AGEs			Inhibition of G.K. peptide-ribose AGEs		
	Concentration (μg/mL)	Inhibition (%)	IC_{50} (μg/mL)	Concentration (μg/mL)	Inhibition (%)	IC_{50} (μg/mL)	Concentration (μg/mL)	Inhibition (%)	IC_{50} (μg/mL)	Concentration (μg/mL)	Inhibition (%)	IC_{50} (μg/mL)
Water extract	100	48.28±1.51	-	300	38.46±0.89	-	100	77.21±0.63	69.87	200	55.73±0.33	161.78
	50	28.78±0.73		200	28.49±0.82		50	31.24±1.72		40	31.73±1.22	
	25	21.55±0.60		100	14.40±1.06		25	10.65±1.31		8	20.72±1.21	
20% Methanol extract	100	53.37±1.34	91.95	300	42.69±1.39	-	100	85.69±0.32	59.43	200	60.33±1.18	140.95
	50	32.81±2.59		200	33.34±1.72		50	44.21±0.83		40	33.81±0.56	
	25	24.19±2.18		100	20.58±1.56		25	17.12±0.75		8	22.64±0.50	
40% Methanol extract	100	52.16±1.71	88.23	300	51.13±0.63	280.46	100	90.79±0.66	41.98	200	67.65±0.93	113.31
	50	39.48±1.00		200	40.90±0.50		50	60.83±0.37		40	36.85±0.29	
	25	25.26±2.16		100	22.55±1.23		25	23.82±0.86		8	26.16±1.15	
60% Methanol extract	100	52.60±1.43	82.60	300	51.33±0.65	276.72	100	91.65±0.15	39.23	200	68.91±0.30	110.42
	50	42.49±0.33		200	42.17±1.22		50	66.30±1.59		40	37.46±0.46	
	25	28.48±2.12		100	24.25±0.31		25	26.71±0.52		8	25.27±1.08	
80% Methanol extract	100	54.62±1.08	75.21	300	51.80±0.27	277.57	100	92.89±0.41	36.97	200	68.59±0.79	109.87
	50	43.62±1.79		200	42.03±0.42		50	70.24±0.38		40	37.91±1.05	
	25	28.32±2.39		100	27.39±1.26		25	29.45±0.74		8	25.90±0.82	
100% Methanol extract	100	53.57±0.87	82.05	300	51.19±0.34	287.79	100	92.10±0.17	38.08	200	65.32±0.62	118.67
	50	41.07±3.99		200	39.10±1.36		50	68.79±1.09		40	37.39±1.26	
	25	28.52±0.44		100	24.60±0.59		25	27.90±1.40		8	25.73±1.43	
Aminoguanidine hydrochloride	200	27.99±2.63	-	200	79.94±0.93	86.33	200	67.95±0.30	113.95	200	49.6±0.62	230.95
	100	21.61±3.03		100	54.34±0.33		100	44.54±1.59		40	17.06±0.13	
	50	10.55±1.70		50	31.23±1.10		50	25.46±1.57		8	1.98±1.357	

Note: Aminoguanidine hydrochloride was used as a positive control. The results were presented as mean ± standard deviations.

Table S2. Antiglycation activities of the 80% methanol extract and its partitions.

Sample	Inhibition of fructosamine formation			Inhibition of BSA-MGO AGEs			Inhibition of BSA-fructose AGEs			Inhibition of G.K. peptide-ribose AGEs		
	Concentration (μg/mL)	Inhibition (%)	IC_{50} (μg/mL)	Concentration (μg/mL)	Inhibition (%)	IC_{50} (μg/mL)	Concentration (μg/mL)	Inhibition (%)	IC_{50} (μg/mL)	Concentration (μg/mL)	Inhibition (%)	IC_{50} (μg/mL)
80% Methanol extract	100	53.57±0.87	82.05	200	43.55±1.16	-	100	92.09±0.20	38.08	1000	95.42±0.05	76.80
	50	41.07±3.99		100	29.30±0.77		50	68.78±1.34		200	68.40±0.82	
	25	28.52±0.44		50	18.71±0.63		25	27.91±0.87		40	37.72±1.42	
Partitioned upper layer	100	55.37±1.42	71.51	200	56.53±0.66	161.14	100	92.43±0.49	37.19	1000	97.15±0.11	58.68
	50	44.70±2.14		100	40.42±0.91		50	68.10±1.62		200	77.37±1.42	
	25	30.78±1.88		50	26.70±1.96		25	30.07±0.37		40	41.22±2.01	
Partitioned lower layer	100	45.93±5.29	-	200	32.43±0.91	-	100	91.10±2.18	40.45	1000	89.95±0.16	96.30
	50	40.43±1.16		100	18.84±0.77		50	65.99±1.62		200	58.62±0.82	
	25	28.90±1.81		50	10.25±1.10		25	24.22±1.00		40	37.57±0.33	

Note: The partitioned upper layer and lower layer samples were obtained by partition of the 80% methanol extract of Pasuchaca by the solvent system ethyl acetate/methanol/water (6:1:5, v/v). The results were presented as mean ± standard deviations.

Table S3. Antiglycation activities of astilbin separated from Pasuchaca.

Sample	Inhibition of fructosamine formation			Inhibition of BSA-MGO AGEs			Inhibition of BSA-fructose AGEs			Inhibition of G.K. peptide-ribose AGEs		
	Concentration (μM)	Inhibition (%)	IC_{50} (μM)	Concentration (μM)	Inhibition (%)	IC_{50} (μM)	Concentration (μM)	Inhibition (%)	IC_{50} (μM)	Concentration (μM)	Inhibition (%)	IC_{50} (μM)
Astilbin	500	60.12±2.09	149.52	1000	65.50±0.62	475.45	250	94.71±0.65	84.29	2000	94.65±0.21	182.60
	250	58.054±0.53		500	51.85±1.18		125	78.93±0.41		400	69.22±0.12	
	125	47.71±1.95		250	35.88±0.98		62.5	33.09±1.43		80	32.26±1.54	
Aminoguanidine hydrochloride	500	13.69±1.12	-	1000	58.74±0.78	816.27	2000	72.37±1.01	1042.56	2000	53.49±0.19	1833.63
	250	9.95±1.47		500	35.40±0.62		1000	46.27±0.52		400	17.43±1.43	
	125	6.87±0.44		250	20.65±0.46		500	27.26±1.35		80	5.73±0.75	

Note: Aminoguanidine hydrochloride was used as a positive control. The results were presented as mean ± standard deviations.

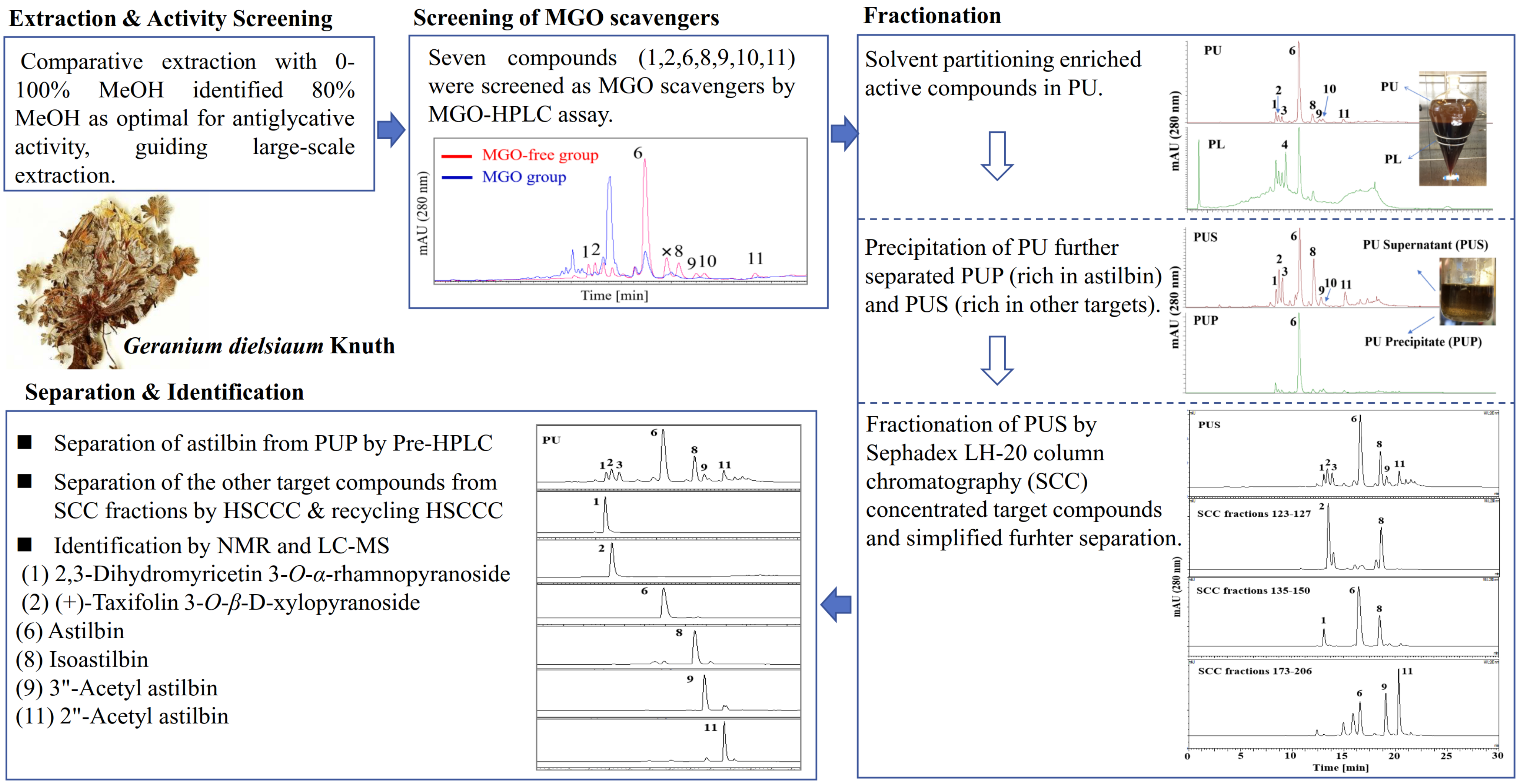

Figure S3. Schematic workflow for the screening, isolation, and identification of antiglycative compounds from Pasuchaca.

Table S4. HPLC validation data for astilbin: calibration curve, LOD, LOQ, and precision.

Parameter		Astilbin
Calibration curve (*n*=3), r^2		y=72494x+178329, r^2=0.9973
Linear range (μg/mL)		2.5-100
Limit of detection (μg/mL, *S*/*N*=3)		0.625
Limit of quantification (μg/mL, *S*/*N*=10)		2.00
Intra-day variability (*n*=6), RSD (%)	25.00 μg/mL	5.34
	70.00 μg/mL	3.59
Inter-day variability (*n*=3), RSD (%)	25.00 μg/mL	5.97
	70.00 μg/mL	5.51

Note: the units of y and x in the calibration curve are uAU and μg/mL, respectively. RSD is the abbreviation of relative standard deviation.
